# SIRT7 inactivation reverses metastatic phenotypes in epithelial and mesenchymal tumors

**DOI:** 10.1038/srep09841

**Published:** 2015-04-29

**Authors:** Shivani Malik, Lidia Villanova, Shinji Tanaka, Misato Aonuma, Nilotpal Roy, Elisabeth Berber, Jonathan R. Pollack, Eriko Michishita-Kioi, Katrin F. Chua

**Affiliations:** 1 Department of Medicine, Division of Endocrinology, Gerontology and Metabolism, School of Medicine, Stanford University, Stanford, California 94305, USA; 2 Geriatric Research, Education, and Clinical Center, VA Palo Alto Health Care System, Palo Alto, California 94304, USA; 3 Department of Experimental Medicine, Sapienza University, Rome, Italy; 4 Venture Science Laboratories, Daiichi Sankyo Co. Ltd., 1-2-58 Hiromachi, Tokyo 140-8710, Japan; 5 Biological Research Department, Daiichi Sankyo RD Novare Co., Ltd., 1-16-13, Kitakasai, Tokyo 134-8630; 6 Diabetes Center, University of California, San Francisco, San Francisco, California 94143; 7 Department of Pathology, Stanford University, Stanford, California 94305, USA

## Abstract

Metastasis is responsible for over 90% of cancer-associated mortality. In epithelial carcinomas, a key process in metastatic progression is the epigenetic reprogramming of an epithelial-to-mesenchymal transition-like (EMT) change towards invasive cellular phenotypes. In non-epithelial cancers, different mechanisms must underlie metastatic change, but relatively little is known about the factors involved. Here, we identify the chromatin regulatory Sirtuin factor SIRT7 as a key regulator of metastatic phenotypes in both epithelial and mesenchymal cancer cells. In epithelial prostate carcinomas, high SIRT7 levels are associated with aggressive cancer phenotypes, metastatic disease, and poor patient prognosis, and depletion of SIRT7 can reprogram these cells to a less aggressive phenotype. Interestingly, SIRT7 is also important for maintaining the invasiveness and metastatic potential of non-epithelial sarcoma cells. Moreover, SIRT7 inactivation dramatically suppresses cancer cell metastasis *in vivo,* independent of changes in primary tumor growth. Mechanistically, we also uncover a novel link between SIRT7 and its family member SIRT1, providing the first demonstration of direct interaction and functional interplay between two mammalian sirtuins. Together with previous work, our findings highlight the broad role of SIRT7 in maintaining the metastatic cellular phenotype in diverse cancers.

SIRT7 is a member of the Sirtuin family of NAD^+^-dependent enzymes, which play diverse roles in aging, metabolism, and disease biology^1^,^2^. Relatively little is understood about SIRT7 function, and only a handful of molecular substrates of SIRT7 have yet been identified. At chromatin, SIRT7 catalyzes selective deacetylation of lysine 18 on histone H3 (H3K18), an emerging epigenetic biomarker of aggressive tumors and poor clinical outcome in cancer patients^3^. Through H3K18 deacetylation at specific promoters, SIRT7 controls a tumor suppressive gene expression program that stabilizes the transformed state of cancer cells. Indeed, inactivation of SIRT7 is sufficient to reverse essential properties of cancer cells, including anchorage-independent growth, loss of contact inhibition, growth in low serum conditions, and tumor growth in mouse xenograft assays^3^.

Studies of SIRT7 expression in human tumor tissues suggest that increased SIRT7 levels may correlate with enhanced tumor aggressiveness^1^. For example, in microarray analyses of human hepatocellular biopsies, SIRT7 expression was found to increase progressively from pre-neoplastic lesions to high-grade tumors^4^. Modest increases in SIRT7 expression have also been detected in thyroid and breast cancer biopsies compared to normal control biopsies, with some correlation with more advanced disease^5^,^6^. Similarly, high SIRT7 expression correlated with advanced tumor stage and decreased overall and disease-free patient survival in colon carcinoma cells^7^. These studies suggested that SIRT7 might play a role in promoting the development of aggressive cancer phenotypes.

Metastasis is the leading cause of cancer-related deaths in the world^8^. The multistep process of invasion and metastasis begins with local invasion, followed by intravasation by cancer cells into nearby blood and lymphatic vessels and transit through the lymphatic and hematogenous systems^8^,^9^. Cancer cells then extravasate from the lumina of such vessels into the parenchyma of distant tissues, where they form micrometastases that can grow and colonize as macroscopic tumors. The process by which neoplastic cells acquire the traits necessary to execute the invasion-metastasis cascade have been primarily studied in the context of epithelial cancers, such as carcinomas, where acquisition of metastatic potential is characterized by the activation of an epithelial-to-mesenchymal transition (EMT)-like program^8^,^10^. During this reversible process, expression of key epithelial maintenance factors such as E-cadherin (CDH1) is suppressed, leading to loss of E-cadherin-mediated cell–cell adhesion and other epithelial traits. Concomitantly, expression of mesenchymal markers and extracellular matrix remodeling enzymes is increased, together with a profound reorganization of the actin cytoskeleton. This phenotypic EMT reprogramming endows cancer cells with the plasticity and motility necessary to undergo the invasion-metastasis cascade. In contrast to the role of EMT in metastatic progression of carcinomas, the mechanisms underlying metastasis of non-epithelial tumors, such as mesenchymal soft tissue sarcomas, are much less understood and are proposed to differ substantially from those in epithelial cancers^11^.

Here, we have uncovered a role for SIRT7 in promoting acquisition of an invasive phenotype in both epithelial and mesenchymal cancer cells. Inactivation of SIRT7 reverses the loss of E-cadherin expression and other associated EMT changes in carcinoma cells, and surprisingly, also attenuates the invasiveness and metastasis of mesenchymal sarcoma cells. Importantly, we use a system in which the role of SIRT7 in regulating the metastatic potential of cancer cells in vivo can be examined without potential confounding effects of SIRT7 on intrinsic primary tumor growth. We also report that SIRT7 interacts functionally with SIRT1, another member of the sirtuin family that has been shown to promote prostate cancer cell migration and metastasis by repressing E-cadherin expression. Together, our findings identify SIRT7 activity as a positive determinant of cancer metastasis, and uncover previously unappreciated crosstalk between two chromatin regulators of the sirtuin family that promote the invasive and metastatic properties of cancer cells.

## Results

### Increased SIRT7 expression and gene amplification is associated with metastatic cancer

**Figure 1:**
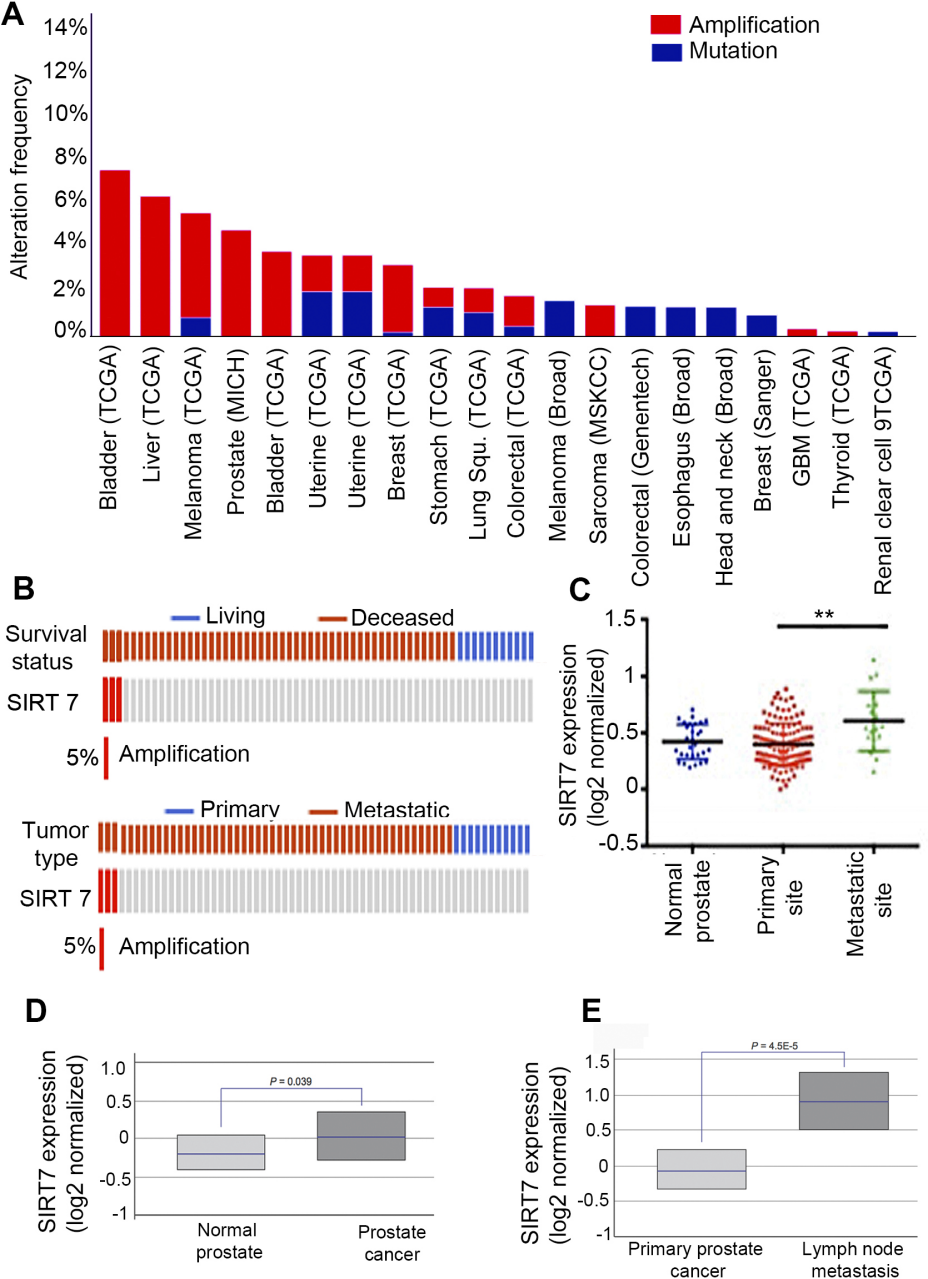
SIRT7 amplification and over-expression is associated with metastatic disease and poor prognosis. (A), Analysis of several cBio Cancer Genomics datasets shows that SIRT7 is amplified in many epithelial cancers and in mesenchymal sarcomas. (B), Analysis of a reported mutational landscape of metastatic prostate cancer shows exclusive amplification of SIRT7 in prostate cancer patients with metastatic disease and poor survival^12^. (C), Meta-analysis of the prostate cancer dataset^13^ using the Oncomine database showing significantly higher expression of SIRT7 in metastatic tumor sites compared to primary tumor or normal prostate tissues. (D), SIRT7 expression in prostate cancer patient tissues versus normal prostate controls. (E), SIRT7 expression in primary prostate cancer tumors versus metastatic tumor tissue. In (D), and (E), levels are log2 normalized values (median, lower and upper quartiles are shown), retrieved from Lapointe et al^14^.

We previously demonstrated that SIRT7 is over-expressed in several patient-matched tumor samples^3^. To further explore the clinical relevance of SIRT7 over-expression, we analyzed human cancer datasets from the cBioportal database for Cancer Genomics. This analysis revealed many human cancer types that harbor amplifications of SIRT7, and a smaller number with mutations at the SIRT7 locus (Fig. 1A). SIRT7 amplifications were by far the predominant genetic change observed in several epithelial cancers (e.g., bladder, liver, prostate and breast carcinomas), as well as in sarcomas. Notably, one study conducted on 61 prostate cancer patients showed amplification of the SIRT7 locus occurring exclusively in tumors that were metastatic and associated with poor survival (Fig. 1B)^12^. In addition, in a human prostate cancer dataset^13^ from the Oncomine gene expression database (http://www.oncomine.com), SIRT7 was significantly over-expressed in metastatic sites compared to the primary tumor sites (Fig. 1C). Moreover, examination of prostate cancer patient microarray expression datasets^14^ revealed that while SIRT7 expression is modestly increased (p = 0.039) in prostate cancer compared to normal prostate tissue controls (Fig. 1D), SIRT7 levels are much more dramatically elevated (p = 4.5e-5) in metastatic lymph node samples compared to primary prostate tumors (Fig. 1E). Together, these data demonstrate that increased SIRT7 expression is specifically associated with human cancer metastasis.

### SIRT7 promotes cancer cell migration and invasiveness

**Figure 2:**
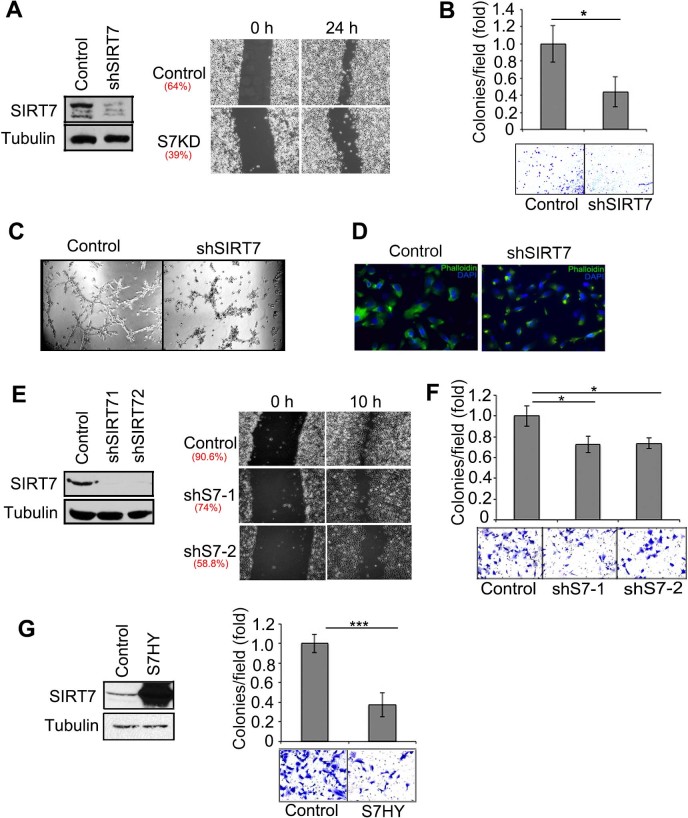
SIRT7 promotes cancer cell migration and invasiveness. (A), Wound healing assay showing reduced migration of SIRT7-depleted (shSIRT7) PC3 cells. (B), Transwell assay showing reduced invasion of SIRT7 depleted PC3 cells. (C), 3D Matrigel assay showing reduced invasive growth of SIRT7 depleted cells through surrounding matrix. Images taken 3 days after plating of cells. (D), Phalloidin staining of F-actin revealed a more collapsed actin cytoskeleton in SIRT7-depleted PC3 cells compared to controls. (E), Impaired cell migration and wound healing in SIRT7- depleted HT1080 cells. (F), Impaired invasion of SIRT-depleted HT1080 cells in Transwell assay. (G), Reduced invasion of HT1080 cells overexpressing the catalytically inactive (S7-HY) SIRT7 protein in the Transwell assay.

To overcome the tissue basement membrane and invade into the stroma, cancer cells must acquire a motile phenotype. To investigate whether SIRT7 regulates the directional migration of cancer cells, we performed wound-healing assays following depletion of SIRT7 by RNA interference (Fig. 2A,E). A wound was created in the cell monolayer and cell migration was monitored using time-lapse microscopy. SIRT7-depletion substantially impaired migration of the metastatic prostate cancer cell line PC3, reducing the healing percentage by ~40% compared to control cells (Fig. 2A). Notably, no significant changes in proliferation were observed under the time frame of these experiments (data not shown).

We next asked whether SIRT7 depletion affects cancer cell invasiveness using two complementary assays. In the Transwell invasion assay, SIRT7-depleted PC3 cells showed reduced invasion through a Matrigel Biocoat^TM^ basement membrane matrix (Fig. 2B). In an independent three-dimensional Matrigel assay, SIRT7-depletion reduced the ability of PC3 cells to invade through the surrounding matrix, as manifested in loss of the branching growth pattern displayed by control cells (Fig. 2C). Consistent with this impaired migration and invasiveness, staining for F-actin (Phalloidin staining) revealed a collapsed actin cytoskeleton upon SIRT7 depletion in PC3 cells (Fig. 2D). Notably, SIRT7 inactivation in the HT1080 sarcoma cell line led to similar reductions in cell migration during wound healing and in Transwell invasion (Fig. 2E, F). These observations demonstrate that inhibiting SIRT7 can reverse metastatic properties of both epithelial and mesenchymal cancer cell types. Moreover, over-expression of a SIRT7 variant bearing a point mutation in the catalytic site (H187Y) also reduced HT1080 cell invasiveness, consistent with a dominant negative effect (Fig. 2G). Together, these results demonstrate that SIRT7 is a positive regulator of cancer cell migration and invasion abilities.

### SIRT7 regulates expression of invasion-related genes and EMT markers in epithelial and mesenchymal cancer cells

To gain additional mechanistic insight into the role of SIRT7 in the invasion-metastasis cascade, we first focused on the effects of SIRT7 inactivation in epithelial carcinoma cells. In these cells, a transition from epithelial to mesenchymal characteristics plays a major role in the acquisition of invasive and motile cellular behavior, and is associated with decreased levels of the epithelial marker and invasion-suppressor factor E-cadherin (*CDH1*), concomitant with *de novo* expression of mesenchymal markers such as the intermediate filament Vimentin^15^,^16^.

**Figure 3:**
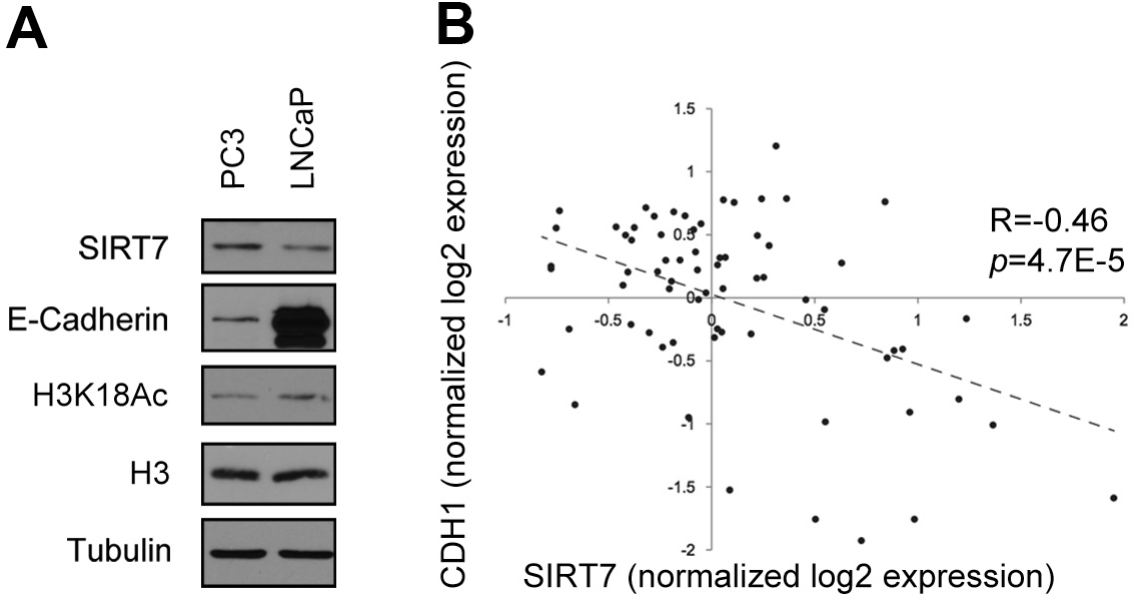
SIRT7 expression correlates inversely with H3K18Ac and E-cadherin in prostate carcinoma. (A), Western blot analysis showing enhanced expression of SIRT7 and lower levels of H3K18Ac and E-cadherin in PC3 cells as compared to LNCaP cells. (B), SIRT7 expression is negatively correlated with E-cadherin (*CDH1*) expression in human prostate cancers. cDNA microarray expression levels are log2 normalized values, retrieved from Lapointe et al^14^. For *CDH1*, the average expression value from 4 cDNA probes was used. Correlation (R) and corresponding P-values are indicated. Western Blots were carried under same experimental conditions. Uncropped images are available in supplementary figure 1a.

Previous studies showed that elevated E-cadherin expression can suppress epithelial tumor cell invasiveness, whereas reduced E-cadherin levels correlate with more aggressive tumor stages and poor clinical outcome^17^,^18^. Similarly, global hypoacetylation of H3K18, the histone substrate of SIRT7, predicts poor prognosis of prostate cancer patients^19^. Consistent with these observations, our western analyses revealed that global levels of E-cadherin and H3K18Ac were substantially lower in the highly aggressive PC3 cell line than in the less aggressive LNCaP cell line, whereas SIRT7 protein showed the opposite pattern (Fig. 3A). Moreover, analysis of large microarray datasets of prostate cancer patient data^14^ revealed a clear inverse correlation between SIRT7 levels and E-cadherin (p = 4.7E-5) (Fig. 3B). These observations suggested that SIRT7 might regulate the expression of E-cadherin or other EMT regulatory factors.

**Figure 4:**
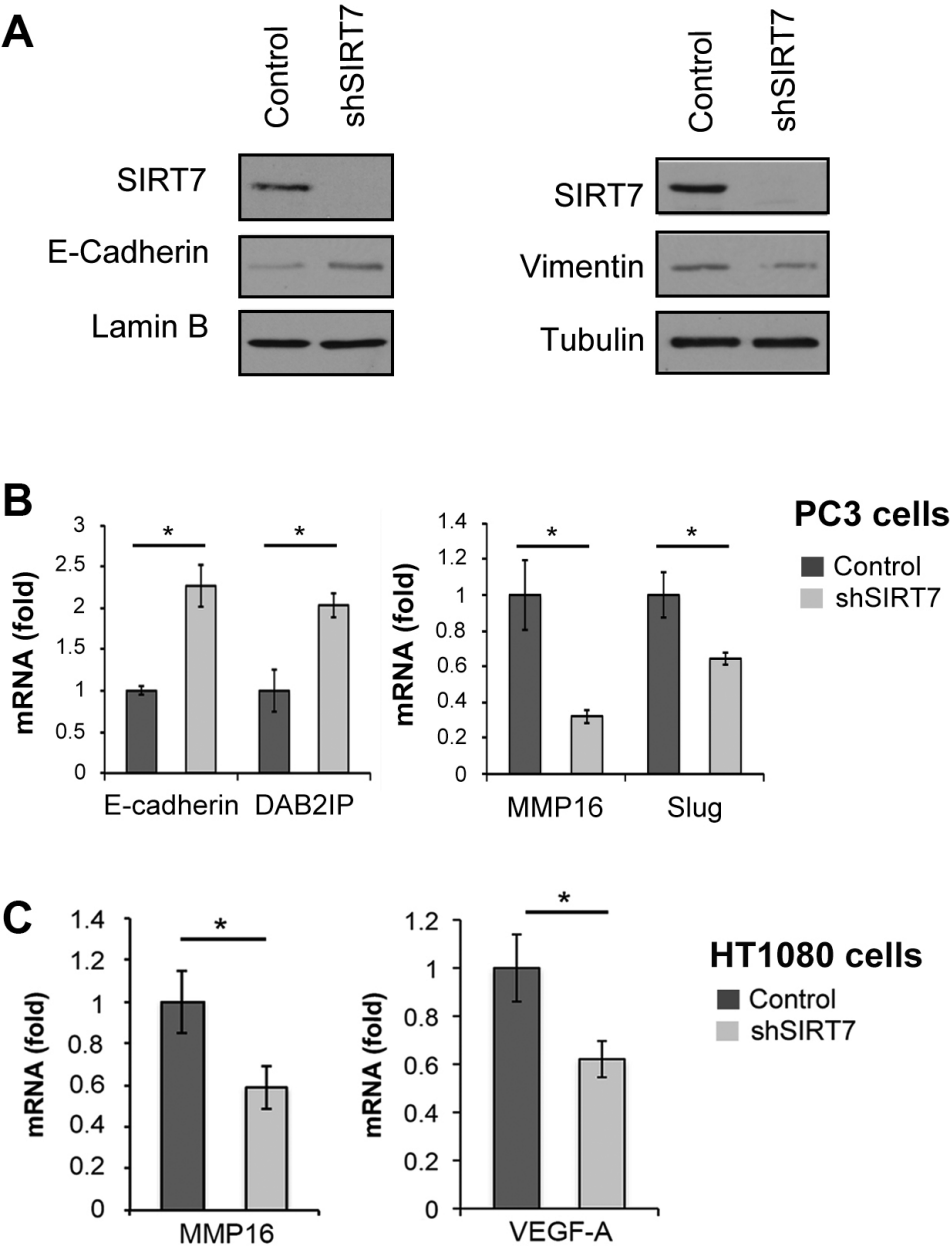
SIRT7 regulates metastasis regulatory genes in epithelial and mesenchymal cancer cells. (A), Western blot analysis showing decreased E-cadherin protein and increased Vimentin levels in SIRT7-deficient PC3 cells (shSIRT7). (B), RT-qPCR analysis showing modulation of E-cadherin, DAB2IP, MMP16, and Slug expression in SIRT7-depleted PC3. (C), RT-qPCR showing decreased expression of MMP16 and VEGF-A in SIRT7 depleted HT1080 cells. Western Blots were carried under same experimental conditions. Uncropped images are available in supplementary figure 1b.

Indeed, western analysis revealed that in SIRT7 deficient PC3 cells, E-cadherin protein levels were up-regulated, and Vimentin protein levels were reduced, consistent with a reversal of the EMT phenotype (Fig. 4A). Next, we examined the mRNA expression levels of several additional markers of EMT. In addition to the up-regulation of E-cadherin mRNA, SIRT7-deficient PC3 cells also showed significantly increased mRNA levels of DAB2IP (DAB2 interacting protein) (Fig. 4B), a tumor suppressor gene whose loss promotes EMT and metastasis in prostate cancer^20^,^21^. At the same time, inactivation of SIRT7 led to down-regulation of the EMT-inducing transcription factor Slug (*SNAI2*) (Fig. 4B), a transcription factor of the Snail family that is one of the central regulators of the EMT program^15^. Thus, the reduced invasiveness of SIRT7-deficient PC3 cells is associated with a reprogramming towards epithelial gene expression, with increased expression of EMT-suppressing genes and down-regulation of EMT-promoting genes.

Importantly, however, our finding that SIRT7 affects the invasive properties of HT1080 sarcoma cells (Fig. 2E–G) suggests that SIRT7 must also impinge on metastasis regulatory pathways that operate in mesenchymal cancers, independent of EMT. Although relatively little is known about the mechanisms or factors involved in metastasis of mesenchymal tumors, recent studies suggest that important players include matrix metalloproteinase (MMP) enzymes as well as the extracellular signaling factor VEGF-A (vascular endothelial growth factor)^11^,^22^. Up-regulation of MMPs is associated with metastatic potential in both epithelial carcinoma and mesenchymal sarcoma tumors^8^,^11^, and endows tumor cells with the ability to breakdown the extracellular matrix and disrupt the basement membrane for tumor cell invasion. We found that SIRT7-inactivation in both PC3 and HT1080 cells led to reduced expression of the matrix metalloproteinase gene *MMP16* (Fig. 4B,C). Moreover, SIRT7-deficient HT1080 cells had reduced expression of VEGF-A (Fig. 4C), which was recently shown to play a crucial role in metastasis of these cells^8^,^22^.

Together, these results suggest that SIRT7 controls the expression of a subset of genes linked to diverse aspects of the invasion-metastasis cascade, including EMT-regulatory factors in epithelial cancers, as well as regulators of cellular invasiveness and extracellular signaling factors in mesenchymal sarcoma cells.

### SIRT7 cooperates with SIRT1 to repress E-cadherin expression

Similar to our findings on SIRT7, previous work showed that the SIRT7 family member SIRT1 can enhance metastatic properties of prostate cancer cells and promote transcriptional repression of E-cadherin^23^,^24^. However, whether these effects of SIRT1 are mediated through its deacetylase activity has not been directly demonstrated, and the biochemical mechanisms underlying SIRT1 regulation of EMT-related genes and metastasis are still unclear. Indeed, the role of the deacetylase activity of SIRT1 in tumor growth and metastasis was recently challenged by observations that the catalytic activity of SIRT1 has little effect on tumor growth and metastasis in a mouse tumor model^25^.

**Figure 5:**
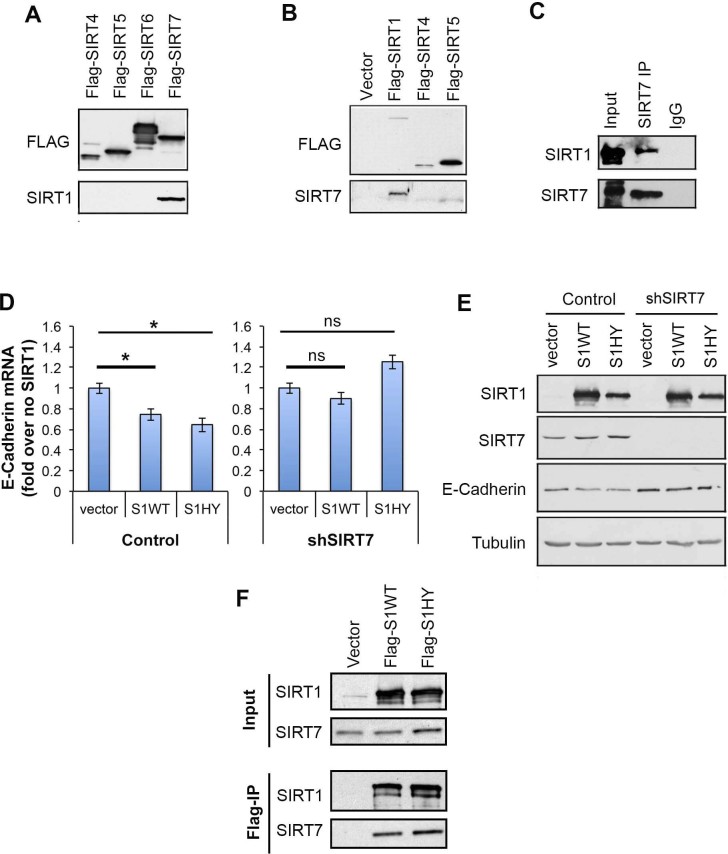
SIRT7 physically interacts with SIRT1 and is required for SIRT1-mediated repression of E-cadherin. (A), Western analysis showing co-immunoprecipitation (IP) of Flag-tagged SIRT7 and endogenous SIRT1. (B), Western analysis showing co-IP of Flag-tagged SIRT1 and endogenous SIRT7. (C), Western analysis showing co-IP of endogenous SIRT1 and SIRT7 proteins. (D), q-PCR analysis of the E-cadherin expression following overexpression of wild-type (S1WT) or catalytically inactive (S1HY) SIRT1 proteins in Control or SIRT7-depleted (shSIRT7) PC3 cells. (E), Western blot showing E-cadherin protein levels from cells used in D. (F), Western blot analysis showing co-immunoprecipitation of SIRT7 by the SIRT1 WT and HY mutant proteins. Western Blots were carried under same experimental conditions. Uncropped images are available in supplementary figure 1c.

The similar roles of SIRT1 and SIRT7 in E-cadherin regulation prompted us to ask if these proteins might intersect in a common mechanism. Consistent with this possibility, we observed that SIRT7 interacts physically with SIRT1 in multiple assays, using both Flag-tagged and endogenous SIRT1 and SIRT7 proteins (Fig. 5A–C). To directly investigate functional interplay between SIRT1 and SIRT7, we asked if SIRT7 influences the ability of SIRT1 to repress E-cadherin expression. Strikingly, RNAi-depletion of SIRT7 completely abolished the previously reported repression of E-cadherin levels by SIRT1 (Fig. 5D, E). Interestingly, our data also revealed that the deacetylase activity of SIRT1 is not required for E-cadherin repression, because a catalytically inactive SIRT1 protein (SIRT1-HY) reduced E-cadherin levels as well as wild-type SIRT1 (Fig. 5D, E). These observations, together with our finding that both wild-type and mutant SIRT1 proteins interact equally with SIRT7 (Fig. 5F), suggest that the physical interaction of SIRT1 with SIRT7 is required for SIRT1-dependent transcriptional repression of E-cadherin in prostate carcinoma cells.

### SIRT7 depletion impairs metastasis *in vivo*

**Figure 6:**
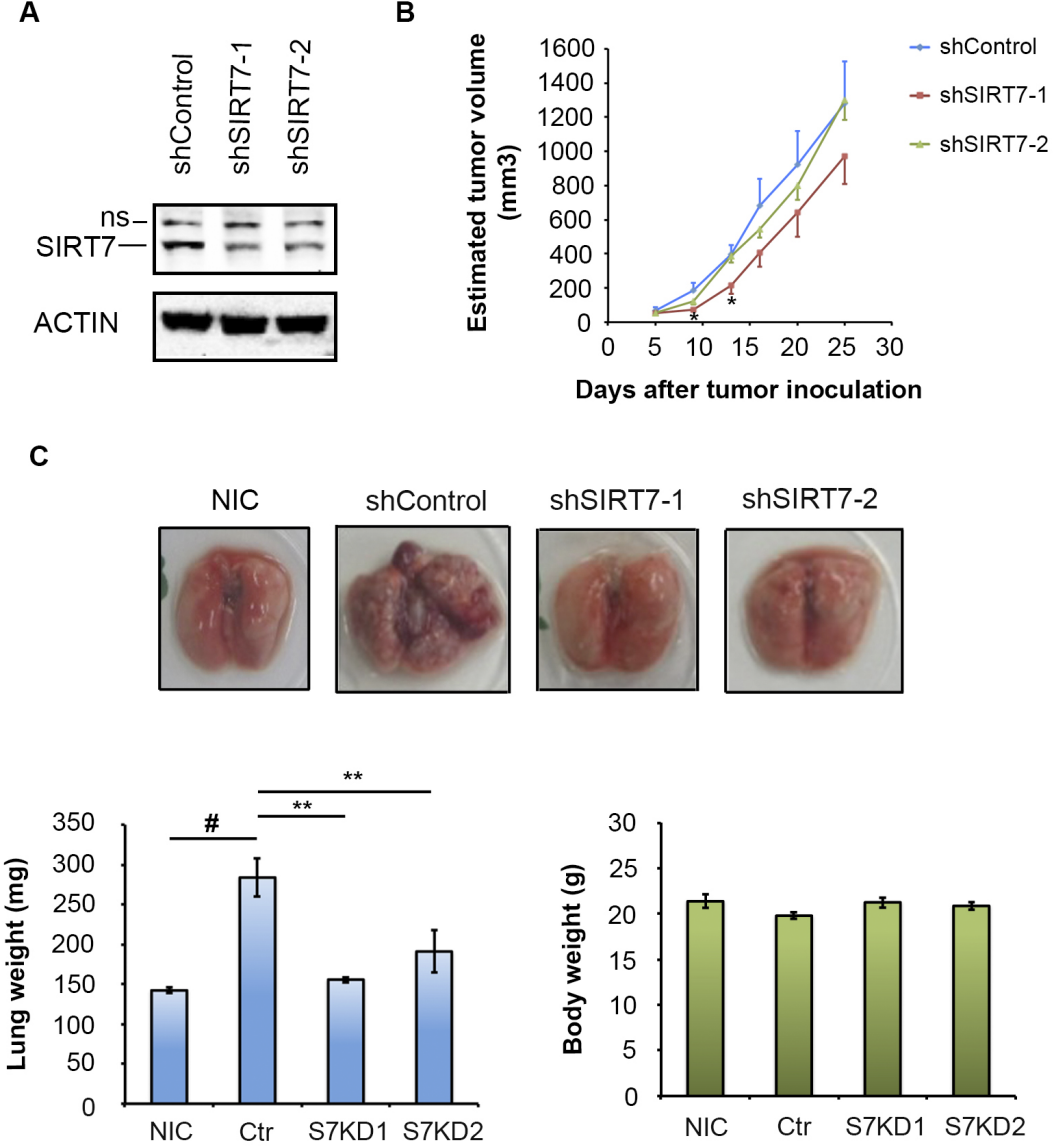
SIRT7 depletion inhibits lung metastasis *in vivo*. (A). Western blot of SIRT7 expression in cells with partial SIRT7 depletion. (B), Tumor volume of subcutaneous xenografts of the SIRT7-depleted and control cells. (C), Effects of SIRT7 depletion on lung metastasis following tail vein injection of immunodeficient mice with control (shcontrol) or SIRT7-depleted (shSIRT7-1, shSIRT7-2) cells. Non-Injected Control (NIC): normal lungs. *, ** indicate significant differences between the sh-control and shSIRT7 groups by Welch's T test (**: P < 0.005, *: P < 0.05). # indicates significant difference between the no injection control lungs and the sh-control HT1080-injected group (P < 0.005). ns, non-specific band.

We previously showed that SIRT7 depletion impairs cancer cell proliferation and reduces primary tumor growth in mouse xenograft assays^3^. In addition to its effects on the primary tumor, the reduced proliferative capacity of SIRT7-deficient cancer cells could also contribute to faster growth of metastatic lesions, even without fundamental changes in metastatic potential. Therefore, to exclude effects of cell proliferation on scoring of metastatic activity, we partially depleted SIRT7 to levels at which little or no significant changes on primary tumor growth were observed (Fig. 6A, B). We then used these cells in a lung metastasis model assay. As expected, tail vein injection of mice with control treated cells led to dramatic metastatic lung lesions. Strikingly, in the SIRT7-depleted cells, the lung metastases were virtually eliminated (Fig. 6C). These findings provide evidence that SIRT7 inactivation in cancer cells specifically prevents metastasis *in vivo*, independent of changes in intrinsic tumor cell proliferative capacity.

## Discussion

Over the past few years, growing evidence has indicated that SIRT7 has important roles in regulating oncogenic transformation and tumor biology. Through deacetylation of histone H3 on lysine K18 at chromatin, SIRT7 controls a tumor suppressive gene expression program that maintains the neoplastic state of cancer cells^3^. SIRT7 also orchestrates several molecular processes – including ribosomal RNA and protein synthesis and ER stress responsiveness – that could be important for cellular changes in cancer^3^,^26^. Consistent with these functions, inactivation of SIRT7 in cancer cells leads to loss of oncogenic properties and reduces the ability of these cells to form tumors in mice^3^,^4^,^7^. In this study, we show that in addition to its effects on primary tumors, inactivation of SIRT7 also has specific metastasis suppressing effects, reducing the migration and invasion of cancer cells *in vitro* and preventing metastasis *in vivo*. Importantly, we also identify SIRT7 as one of few known regulators of metastatic change in mesenchymal cancers, for which candidate therapeutic targets are greatly needed^27^.

Our findings fit well with recent evidence that SIRT7 depletion reduces metastasis by colorectal cancer cells in mouse xenograft metastasis models^7^. Our experiments extend these findings to non-epithelial soft tissue sarcoma cancers, and importantly, also exclude the possibility that the observed decrease in metastatic lesions might be due to changes in oncogenic properties more generally, rather than metastatic cell properties per se. Specifically, we use a system in which SIRT7 depletion is titrated to levels where primary tumor growth is minimally or not affected, and yet metastasis by these cells is virtually abolished.

Recent analyses of human cancer patient microarray datasets have detected elevated levels of SIRT7 in hepatocellular and colorectal carcinomas, with higher SIRT7 levels correlating with increased disease severity^4^,^7^. In this study, we have added to the human cancer genomics analyses in several ways. First, we show that while SIRT7 expression is higher in tumor compared to normal control tissues, SIRT7 levels are even more dramatically elevated in metastatic tissue compared to primary tumors. Second, we detect a significant enrichment for SIRT7 amplifications in a majority of tumor types that we analyzed, including carcinomas of many tissue origins, and non-epithelial soft tissue sarcomas. By contrast, little amplification of SIRT1 or SIRT6 was observed in similar analyses (data not shown). Moreover, we show that in a set of prostate carcinoma samples, the SIRT7 amplifications are selectively detected in metastatic tumors with poor clinical outcome, and not observed in primary tumors with better patient survival. Finally, we demonstrate a strong inverse correlation between levels of SIRT7 and E-cadherin, a marker of aggressive tumor stages and poor clinical prognosis^17^,^18^.

We have uncovered a novel physical and functional interaction between SIRT7 and SIRT1 in controlling E-cadherin expression. Based on our findings that the catalytic activity of SIRT1 is dispensable for E-cadherin repression, whereas SIRT7 is required for E-cadherin repression, we hypothesize that SIRT1 might function as a scaffold protein in recruiting SIRT7 to the E-cadherin promoter for SIRT7-mediated deacetylation of H3K18Ac. However, our data do not rule out alternative mechanistic interactions between these two sirtuins. The requirement for SIRT7 in SIRT1-dependent EMT promotion might also be relevant for understanding the observation that in contrast to the EMT- and metastasis- promoting effects of SIRT7 reported in prostate cancer cells^23^, other work reported that in breast cancer cells, SIRT7 suppresses EMT and tumor metastasis via deacetylation of the Smad4 transcription factor^28^. These discrepant results were attributed to potential cell-type specific mechanisms, and our data suggest that these might involve differential interaction of SIRT1 with SIRT7 in the different cell contexts.

Our findings of functional interaction between SIRT1 and SIRT7 also suggest that similar interplay might operate in additional physiologic contexts. For example, increased acetylation of the tumor suppressor p53 has been reported in SIRT7-deficient hearts^29^, but little deacetylation activity of p53 by SIRT7 was observed in vitro or in cultured cells^3^,^29^,^30^. Since SIRT1 is a robust p53 deacetylase, we speculate that SIRT7 levels might influence p53 acetylation indirectly through its functional interaction with SIRT1, and this might be biologically limiting in the context of the SIRT7 mouse knockout hearts but not some other settings. SIRT1 and SIRT7 are also both implicated in regulating rDNA transcription in nucleoli but with opposite effects^31^,^32^,^33^. It is possible that these functions might also be linked via interaction of SIRT1 and SIRT7 in nucleoli.

Much evidence implicates regulation of protein acetylation and deacetylation in diverse aspects of tumor biology, and inhibitors of non-sirtuin histone deacetylase enzymes are already being intensively tested for anti-cancer therapy. Our observations that high SIRT7 expression is associated with aggressive metastatic phenotypes of human tumors, and that SIRT7 depletion significantly impairs both primary tumor growth and metastatic spread in mouse tumor models, suggest that pharmacologic targeting of SIRT7 might be a promising therapeutic strategy, and could be used in the context of combinatorial therapy with other HDAC inhibitors. Thus, our findings prompt future investigations into the roles of SIRT7 to exploit its use as a potential therapeutic target in advanced cancer stages.

## Methods

### Cell Culture

Cells were grown in a humidified tissue culture incubator, at 37°C, 5% CO_2_ atmosphere. LNCaP and PC3 cells were maintained in Dulbecco's Modified Eagle Medium (DMEM) supplemented with 10% FBS, 1% penicillin-streptomycin and 1% L-glutamine (Gibco, Invitrogen). HT1080 cells were grown in Advanced DMEM (Gibco, Invitrogen) in the presence of 10% FBS and 1% penicillin-streptomycin. All cells were obtained from the American Type Culture Collection (Manassas, Virginia).

### Plasmids

Lentiviral plasmids pSicoR-puro encoding shRNAs targeting SIRT7 mRNA were generated as previously described^3^. Target sequences were as follows: S7KD1, 5’-CACCTTTCTGTGAGAACGGAA-3′; S7KD2, 5’-TAGCCATTTGTCCTTGAGGAA-3′. The pBabe-puro retroviral vectors for expression of human wild-type and H187Y SIRT7 proteins were previously described^3^. Flag-tagged-SIRT1 WT and H355Y mutant expression vectors were described in^34^.

### Transwell Invasion Assay

The assay was performed according to BD BioCoat^TM^ manufacturer’s instructions. Briefly, medium containing 10% FBS as a chemoattractant was added to the wells of a 24-well plate. The Matrigel invasion chambers were transferred to the wells containing the chemoattractant using sterile forceps. A suspension of 10^4^ HT1080 cells and 5*10^4^ PC3 cells in serum-free media was loaded into the chambers. Cells were incubated for 22–24hr in a humidified tissue culture incubator, at 37°C, 5% CO_2_ atmosphere. The non-invading cells were removed by scrubbing with a cotton tipped swab and the invading cells were fixed with methanol for 2 minutes and stained with crystal violet for 10 minutes. The inserts were dried and the membrane was photographed through the microscope at 10X magnification.

### Wound Healing Assay

Cells were grown to confluent monolayers. A scratch was then made using 200 uL tip on cell monolayers. Cells were washed once each with PBS and warm media. Cells were imaged immediately after creating the wound using a time lapse microscope every 15 minutes for 12 hr for HT1080 cells and 20 hr for PC3 cells.

### 3D Matrigel Assay

LDEV free Matrigel basement matrix (BD Biosciences, now Corning) was spread evenly on the bottom of chamber slides and allowed to solidify at 37°C for 30 minutes. Control and SIRT7 KD PC3 cells were suspended in 2% matrigel and spread over the matrix. Cells were fed every 3 days with fresh 2% matrigel. Images were taken every 2 days.

### Phalloidin staining

Cells were fixed with 4% paraformaldehyde for 10 minutes and permeabilized with 0.1% with Triton-X for 5 minutes. Cells were stained with 1:100 dilution of the F-actin probe Alexa Fluor 488 Phalloidin (Life Technologies). Images were acquired on a fluorescence microscope using a 20x objective.

### Co-immunoprecipitation and western blot

Cell lysates were prepared as described previously. 1 mg of total protein was used for immunoprecipitation with SIRT7 antibody overnight. The immunoprecipitated complex was incubated with Protein A/G beads (Sigma) for 1 hour followed by washing with 250 mM NaCl containing wash buffer 6 times. The beads were incubated at 37°C for 20 minutes and the supernatant was used for western blot analysis. Flag-co-immunoprecipitation was carried out by using ANTI-FLAG M2 affinity gel (A2220-10 ML, Sigma).

### Antibodies

E-cadherin antibody (610181) was purchased from BD Biosciences, SIRT1 (07-131) and β-tubulin (05–661) antibodies were purchased from Millipore. Vimentin antibody (V5255) was purchased from Sigma. The rabbit polyclonal antibody specifically recognizing SIRT7 was raised against the following synthetic peptide GWFGRGCTKRTKRKKVT and the affinity-purified antibody was used in this study (ref. Michishita E, 2005). Acetylated lysine 18 of histone H3 (H3K18Ac) antibody (ab1191) was purchased from Abcam and Lamin B antibody (C20) from Santa Cruz Biotechnology.

### RT-qPCR

Total RNA was extracted from cells using the RNeasy kit (Qiagen) and reverse transcribed using SuperScript III (Invitrogen) and oligo(dT) primers, according to the manufacturers' instructions. Quantitative real-time PCR analysis was performed on a Roche LightCycler 480 using the manufacturer's SYBR Green system. PCR primers used are:

E-cadherin: Forward 5’-GGTCTGTCATGGAAGGTGCT-3’; Reverse 5’-GATGGCGGCATTGTAGGT-3’

DAB2IP: Forward 5’-TGGACGATGTGCTCTATGCC-3’; Reverse 5’-GGATGGTGATGGTTTGGTAG-3′

MMP16: Forward 5'-ATGCAGCAGTTCTATGGCATT-3'; Reverse 5'-CTGGTCAGGTACACCGCA TC -3'

Slug: Forward 5’-TGTTGCAGTGAGGGCAAGAA-3’; Reverse 5’-GACCCTGGTTGCTTCAAGGA-3′

VEGF-A Forward 5’- CTACCTCCACCATGCCAAGT-3’; Reverse 5’-GCAGTAGCTGCGCTGATAGA-3’

GAPDH: Forward 5’-AGCCACATCGCTCAGACAC-3’; Reverse 5’-GCCCAATACGACCAAATCC-3’

### In vivo xenograft tumor and metastasis studies

Lung metastasis was analyzed following tail-vein injection of 1×10^6^ control or SIRT7-deficient HT1080 cells into SCID mice. Lungs were harvested, weighed and imaged after 28 days. Sub-cutaneous xenograft primary tumor assays were performed as previously described^3^.

## Author Contributions

S.M. and L.V. carried out the experiments and prepared Figs. 2–5. S.M., and N.R. analyzed the data in Fig. 1A–C. S.T. and M.A. performed the experiments in Fig. 6, which were designed and interpreted by E.M.-K. J.R.P. analyzed the data and prepared Figs. 1D, E, and 3A. E.B. contributed to experiments in Fig. 5. K.F.C., S.M., and L.V. wrote the manuscript with input from E.M.-K.

## Supplementary Material

Supplementary Information Supplementary Figures
