# Supplementary material for: SIRT7 inactivation reverses metastatic phenotypes in epithelial and mesenchymal tumors
Source: Sci Rep. 2015 Apr 29;5:9841. doi: 10.1038/srep09841 (PMC4413894; doi:10.1038/srep09841)

Supplementary Information

SIRT7 inactivation reverses metastatic phenotypes in epithelial and mesenchymal tumors

Shivani Malik[1,2,8]*, Lidia Villanova[1,2,3,9]*, Shinji Tanaka[4], Misato Aonuma[5], Nilotpal Roy[6], Elisabeth Berber[1, 2], Jonathan R. Pollack[7], Eriko Michishita-Kioi[4], Katrin F. Chua[1,2]

1 Department of Medicine, Division of Endocrinology, Gerontology and Metabolism, School of Medicine, Stanford University, Stanford, California 94305, USA,

2 Geriatric Research, Education, and Clinical Center, VA Palo Alto Health Care System, Palo Alto, California 94304, USA,

3 Department of Experimental Medicine, Sapienza University, Rome, Italy,

4 Venture Science Laboratories, Daiichi Sankyo Co. Ltd., 1-2-58 Hiromachi, Tokyo 140-8710, Japan,

5 Biological Research Department, Daiichi Sankyo RD Novare Co., Ltd., 1-16-13, Kitakasai, Tokyo 134-8630,

6 Diabetes Center, University of California, San Francisco, San Francisco, California 94143

7 Department of Pathology, Stanford University, Stanford, California 94305, USA

8 Present Address: Department of Medicine, UCSF School of Medicine, San Francisco CA 94158

9 Present address: Department of Hematology, Oncology and Molecular Medicine, Istituto Superiore di Sanita, Rome, Italy

*These authors contributed equally to this work

Correspondence and requests for materials should be addressed to K.F.C. (kfchua@ stanford.edu) or E.M.-K. (kioi.eriko.ur@ daiichisankyo.co.jp)

Supplementary Figure 1. Full length blots for data in Figure 2A,E,G

A

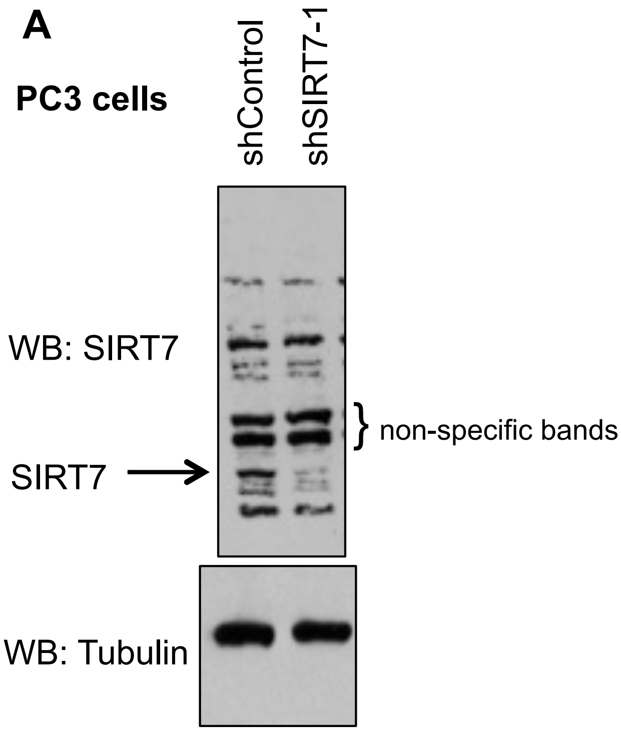

E

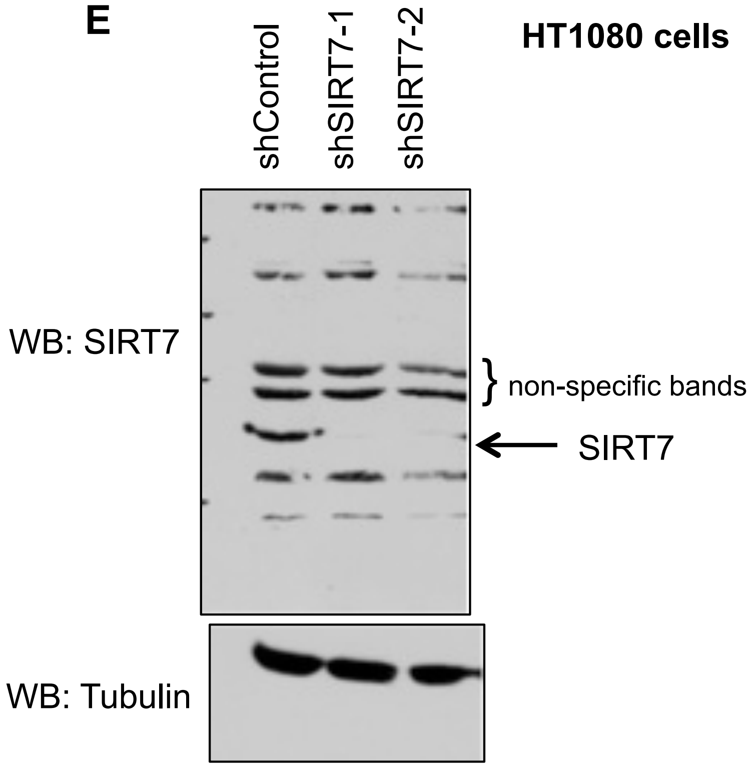

G

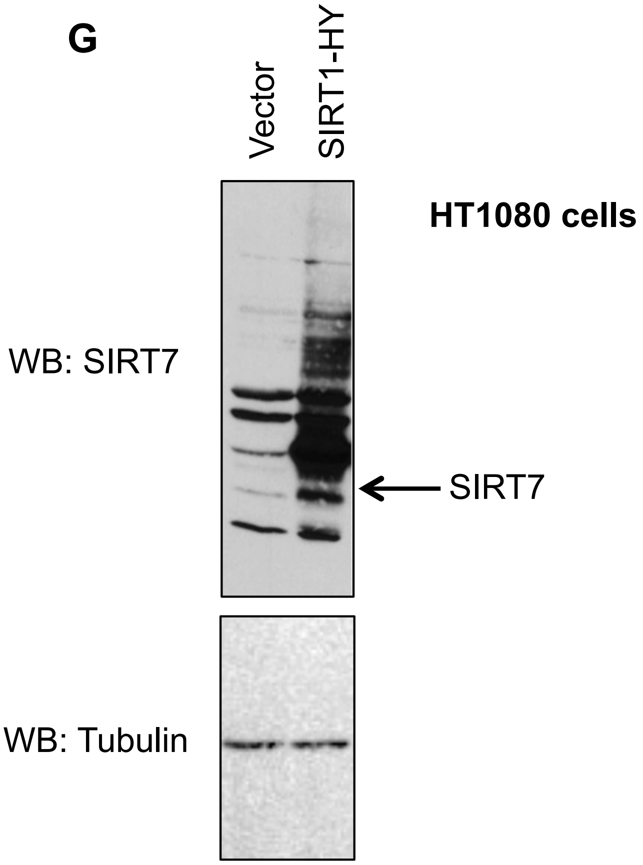

Supplementary Figure 2. Full length blots for data in Figure 3A

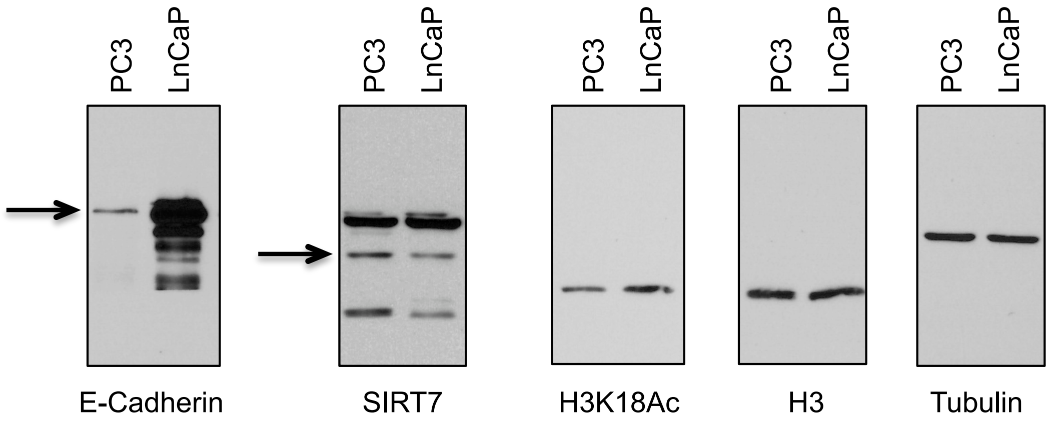

Supplementary Figure 3. Full length blots for data in Figure 4A

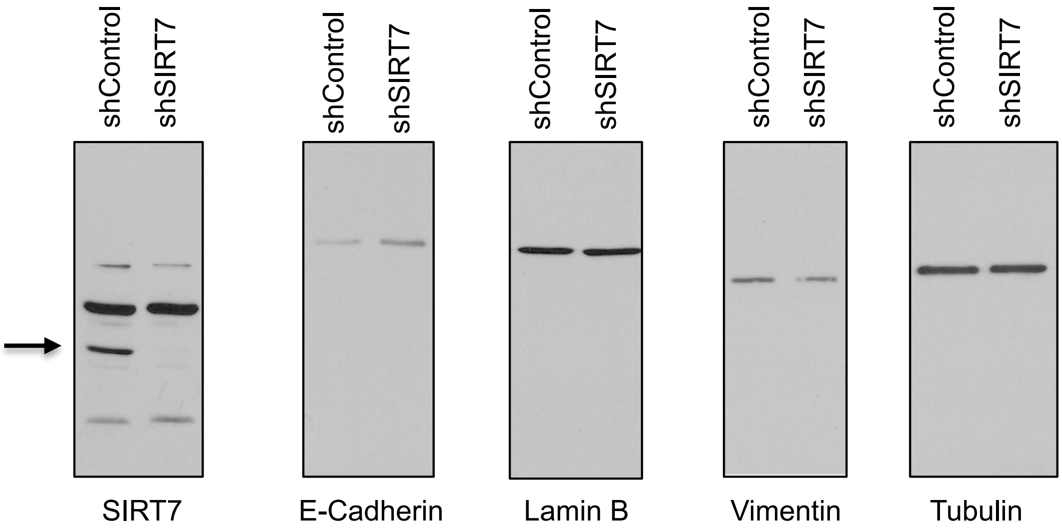

Supplementary Figure 4. Full blots for data in Figure 5A, B, C, E

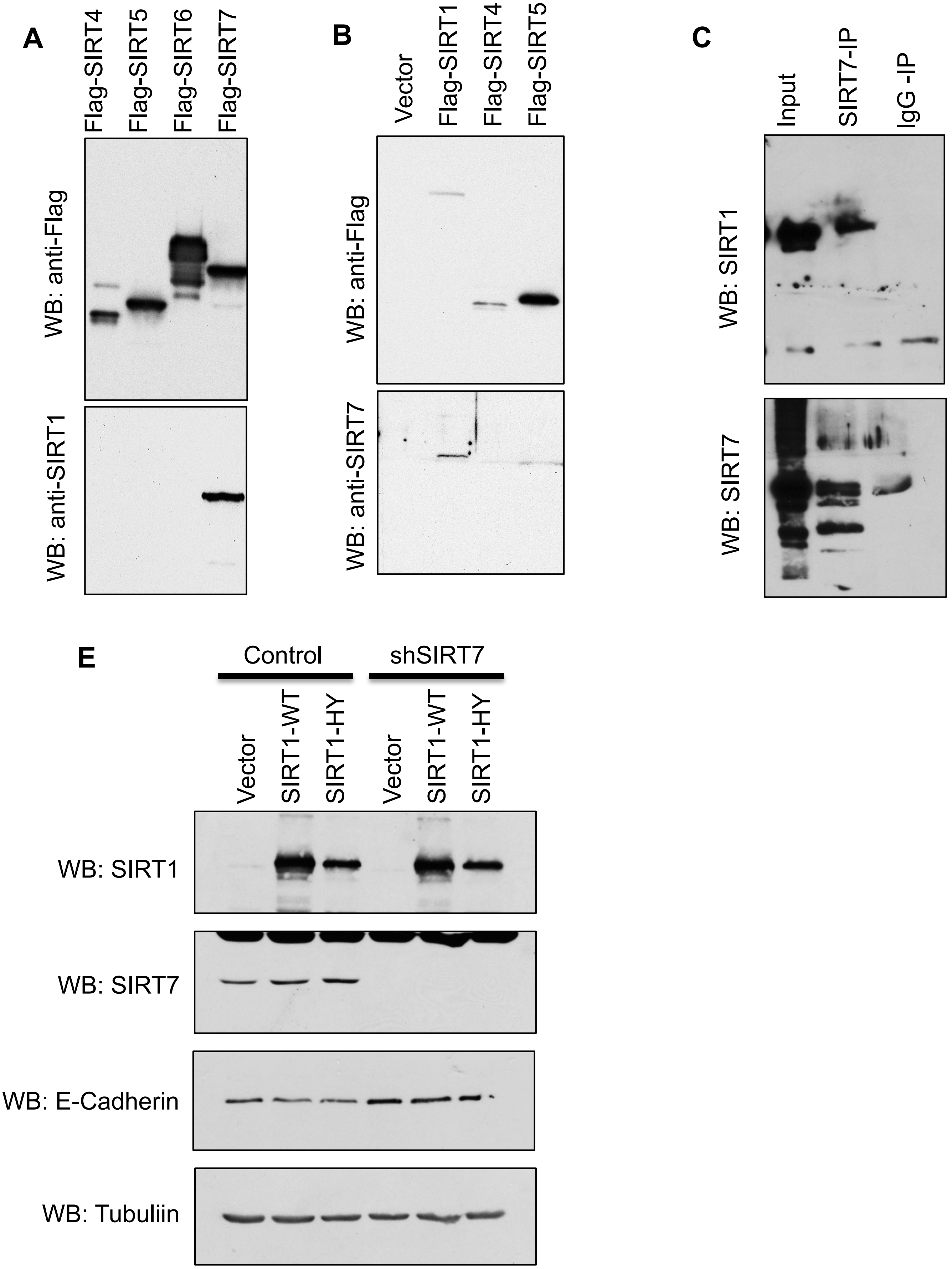

Supplementary Figure 5. Full blots for data in Figure 5F

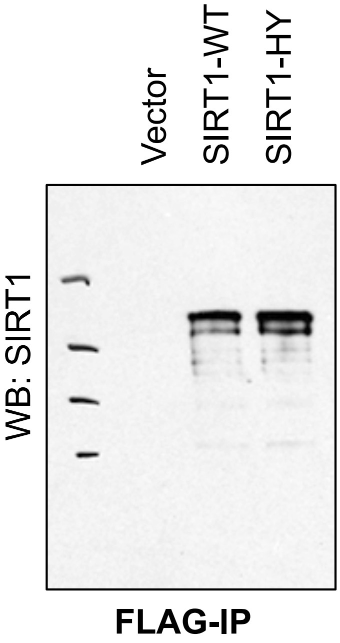

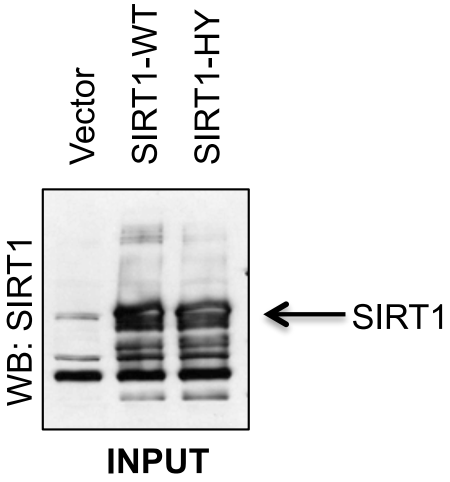

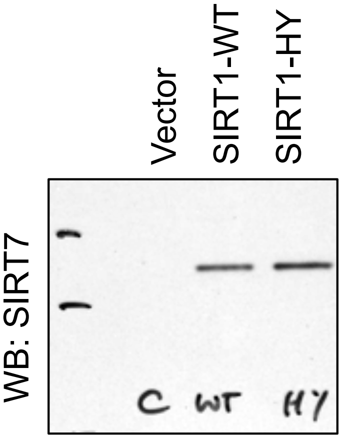

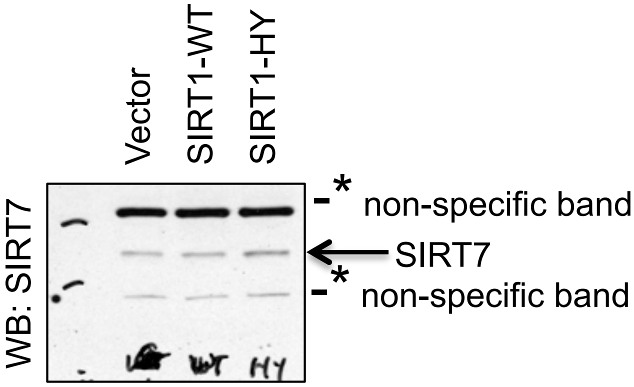

Supplementary Figure 6. Full blot for data in Figure 6.

A

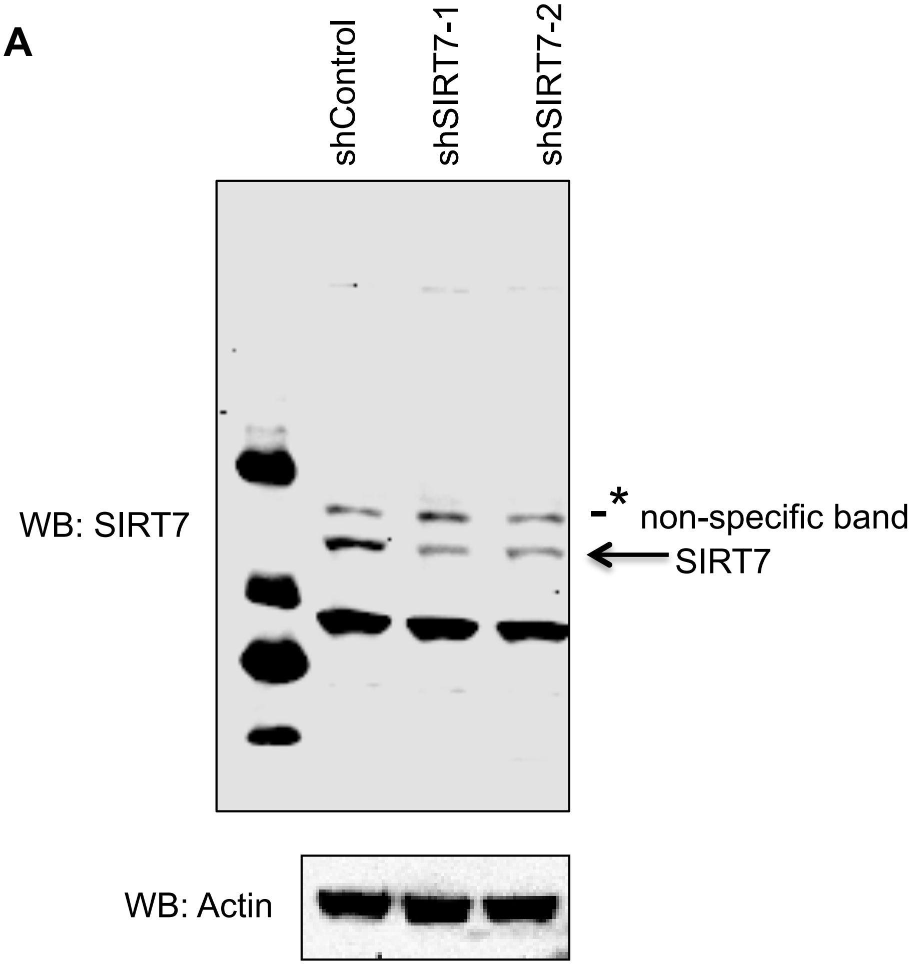

Supplement: Supplementary Information — Supplementary Figures [file srep09841-s1.pdf]
